# METTL3 mediates Ang-II-induced cardiac hypertrophy through accelerating pri-miR-221/222 maturation in an m6A-dependent manner

**DOI:** 10.1186/s11658-022-00349-1

**Published:** 2022-07-14

**Authors:** Rui Zhang, Yangyang Qu, Zhenjun Ji, Chunshu Hao, Yamin Su, Yuyu Yao, Wenjie Zuo, Xi Chen, Mingming Yang, Genshan Ma

**Affiliations:** Department of Cardiology, Zhongda Hospital, School of Medicine, Southeast University, 87 Hunan road, Nanjing, 210000 Jiangsu People’s Republic of China

**Keywords:** Cardiac hypertrophy, Angiotensin II, METTL3, miR-221/222, Wnt/β-catenin signaling

## Abstract

**Background:**

METTL3 is the core catalytic enzyme in m6A and is involved in a variety of cardiovascular diseases. However, whether and how METTL3 plays a role during angiotensin II (Ang-II)-induced myocardial hypertrophy is still unknown.

**Methods:**

Neonatal rat cardiomyocytes (NRCMs) and C57BL/6J mice were treated with Ang-II to induce myocardial hypertrophy. qRT-PCR and western blots were used to detect the expression of RNAs and proteins. Gene function was verified by knockdown and/or overexpression, respectively. Luciferase and RNA immunoprecipitation (RIP) assays were used to verify interactions among multiple genes. Wheat germ agglutinin (WGA), hematoxylin and eosin (H&E), and immunofluorescence were used to examine myocardial size. m6A methylation was detected by a colorimetric kit.

**Results:**

METTL3 and miR-221/222 expression and m6A levels were significantly increased in response to Ang-II stimulation. Knockdown of METTL3 or miR-221/222 could completely abolish the ability of NRCMs to undergo hypertrophy. The expression of miR-221/222 was positively regulated by METTL3, and the levels of pri-miR-221/222 that bind to DGCR8 or form m6A methylation were promoted by METTL3 in NRCMs. The effect of METTL3 knockdown on hypertrophy was antagonized by miR-221/222 overexpression. Mechanically, Wnt/β-catenin signaling was activated during hypertrophy and restrained by METTL3 or miR-221/222 inhibition. The Wnt/β-catenin antagonist DKK2 was directly targeted by miR-221/222, and the effect of miR-221/222 inhibitor on Wnt/β-catenin was abolished after inhibition of DKK2. Finally, AAV9-mediated cardiac METTL3 knockdown was able to attenuate Ang-II-induced cardiac hypertrophy in mouse model.

**Conclusions:**

Our findings suggest that METTL3 positively modulates the pri-miR221/222 maturation process in an m6A-dependent manner and subsequently activates Wnt/β-catenin signaling by inhibiting DKK2, thus promoting Ang-II-induced cardiac hypertrophy. AAV9-mediated cardiac METTL3 knockdown could be a therapeutic for pathological myocardial hypertrophy.

**Supplementary Information:**

The online version contains supplementary material available at 10.1186/s11658-022-00349-1.

## Background

Hypertension is a major risk factor for morbidity and mortality of cardiovascular diseases. The heart’s left ventricle is the primary target for hypertension damage among all the organs. The left ventricle undergoes geometrical changes from concentric remodeling to concentric or eccentric left ventricle hypertrophy, which are independent risk factors for cardiovascular morbidity and mortality [1]. Besides, without intervention, this pathological cardiac hypertrophy progresses to heart failure with preserved/reduced ejection fraction, reducing patients’ quality of life and prognosis [2]. The formation of left ventricle hypertrophy is predominantly due to increased cardiomyocyte size. It is characterized by an overall enhancement in protein synthesis and fetal gene expression, including atrial natriuretic peptide (ANP), brain natriuretic peptide (BNP), and β-myosin heavy chain (βMHC) [3]. In the cardiovascular system, the renin–angiotensin system (RAS) is a key player in controlling homeostasis, and angiotensin (Ang) II is one of its main functional components. As a vasopressor, Ang-II is usually elevated in hypertension, leading to the deterioration of the pressure. Besides, Ang-II acts directly on the myocardium to promote pathological cardiac hypertrophy and cardiac failure [4, 5].

*N*^6^-methyladenosine (m6A) is one of the most abundant internal RNA modifications among the hundreds of post-transcriptional modifications in cellular RNA, mainly appearing in the “A” of the conservative RRACH sequence (R = A or G, H = A, C, or U) [6, 7]. This modification is reversible, and it is catalyzed by the methyltransferase complex, including METTL3, METTL14, and WTAP, and reversed by demethylase FTO and ALKBH5 [7]. The m6A modification has been revealed to affect the splicing, exportation, localization, translation, and stability of target mRNAs, thereby functioning in a variety of diseases such as cancers, neuronal disorders, and psychiatric disorders [8]. Recently, it was shown that METTL3 is increased in cardiomyocytes treated with Ang-II and that USP12 is responsible for promoting the transcription of METTL3 by stabilizing and upregulating p300. METTL3 acted as a pathogenic factor and mediated the pro-hypertrophy effect of USP12 in Ang-II-induced myocardium remodeling [9]. However, the specific mechanism of METTL3 function in Ang-II-induced cardiac hypertrophy is still unclear.

Noncoding RNAs, including miRNAs, are widely involved in cardiovascular diseases, especially in cardiac hypertrophy [10]. The miRNA cluster miR-221/222, consisting of miR-221-3p and miR-222-3p, are both located on chromosome X (Xp11.3) with highly homologous and shared seed sequences [11]. Previous research found that the expression of miR-221/222 was elevated in the hypertrophic myocardium of an Ang-II-induced hypertrophy mouse model [12]. Besides, overexpression of miR-221 could promote cardiac hypertrophy in vitro [13]. Additionally to mRNA, miRNAs were also under the control of m6A modification. It has been reported that METTL3-dependent m6A accelerated maturation of pri-miR-221/222 through interaction with the microprocessor protein DGCR8 [14, 15]. Therefore, whether METTL3 promotes hypertrophy through the miR-221/222-mediated signaling pathway is worth exploring.

In the present research, we found that increased METTL3, coinciding with miR-221/222, mediated the cardiac hypertrophy caused by Ang-II. METTL3-dependent m6A modification was indeed an activator of the pri-miR-221/222 transforming to mature miR-221/222 in cardiomyocytes. Furthermore, DKK2, a target of miR-221/222, was directly inhibited and the Wnt/β-catenin was subsequently activated, thus promoting pathological hypertrophy. Finally, adeno-associated virus (AAV)-9-mediated cardiac METTL3 knockdown in vivo could effectively improve Ang-II-induced myocardial remodeling.

## Materials and methods

### Animals

Sprague-Dawley (SD) rats aged 1–3 days and male C57BL/6J mice aged 5 or 9 weeks were purchased from QingLongShan Animal Breeding Field (Jiangsu, China) and used in this study. The animals were housed in a specific pathogen-free environment under a 12-h light/dark cycle and could freely access food and water.

### Myocardial hypertrophy model in vivo

The mice aged 9 weeks were randomly divided into myocardial hypertrophy or control group (*n* = 5 per group). Myocardial hypertrophy in mice was induced by chronic infusion of Ang-II (MCE) at a rate of 1000 ng/kg/min for 3 weeks using osmotic minipumps (Alzet Model 2004), and the control group mice were infused with saline at the same dose. The operation was performed as previously described [16]. In brief, the pump was prefilled with Ang-II or saline and then incubated in sterile saline at 37 °C for 48 h. After the mice were anesthetized with 3.0% isoflurane mixed with oxygen, an incision was made on the back skin of the mice, and the pump was implanted into the subcutaneous area, followed by suturing the incision. After the operation, the mice were given buprenorphine (0.1 mg/kg) to reach analgesia. Finally, the regaining consciousness mice were returned to cages and fed until the end of the experiment.

To explore the function of METTL3 in vivo, an AAV9 vector containing the short-hairpin RNA (shRNA) targeting METTL3 (shMETTL3) or negative control (shNC) was purchased from GeneChem and used to inhibit METTL3 expression in hearts. The sequence of shMETTL3 is listed in Additional file 1: Table S1. Briefly, 5-week-old mice were randomly divided into three groups: AAV9-shNC + saline (*n* = 7), AAV9-shNC + Ang-II (*n* = 8), AAV9-shMETTL3 + Ang-II (*n* = 7). Each mouse was injected with the AAV-9 vector containing 2.5 × 10^11^ viral genome particles via tail vein. After 4 weeks, the pumps containing saline or Ang-II were implanted according to the above procedure for 3 weeks to induce cardiac remodeling.

### Echocardiography and histopathological examination

After 3 weeks of Ang-II infusion, the mice were anesthetized with 1.0% inhaled isoflurane and cardiac echocardiography was performed using an ultra-high-resolution small animal ultrasound imaging system (VisualSonics Vevo 3100) to evaluate the structural parameters and cardiac function. The data were analyzed from three consecutive cardiac cycles of each measurement. Several indicators, including left ventricular anterior wall thickness (LVAW), LV ejection fraction (EF, %), and LV fractional shortening (FS, %), were collected. Subsequently, the mice were euthanized to remove hearts, and body weight (BW) and heart weight (HW) were obtained to calculate the HW:BW ratio. Furthermore, the widest part of the middle ventricular tissue of hearts was collected and fixed in 4% paraformaldehyde overnight, embedded in paraffin, and cut into sections of 5 μm thickness. Then the sections were stained with hematoxylin and eosin (H&E, Sigma) to observe the general morphology. The sections were incubated with wheat germ agglutinin (WGA, Sigma) to evaluate the cross‐sectional areas of cardiomyocytes.

### Cell culture and treatment

Primary neonatal rat cardiomyocytes (NRCMs) were isolated from the hearts of 1–3-day-old SD rats according to a previously described method [17]. In brief, the hearts were washed and minced in ice-cold Hank’s balanced salt solution (Gibco) and digested with a mixture of 0.03% trypsin (Hyclone) and 0.04% collagenase type II (Sigma) to isolate cardiomyocytes. Then, cardiac fibroblasts were removed using differential attachment technique, and NRCMs were seeded onto six‐well culture plates and cultured in Dulbecco’s modified Eagle medium (DMEM)/F12 medium (Hyclone) supplemented with 10% fetal bovine serum (FBS, Gibco) and 100 U/mL penicillin/streptomycin (Gibco) at 37 °C with 5% CO_2_ at a density of 2 × 10^5^ cells per well. The myocardial hypertrophy model in vitro was induced by Ang-II (MCE) at a concentration of 400 nmol/L for 48 h.

HEK293T cells were purchased from the Cell Bank of the Chinese Academy of Sciences (Shanghai, China), and cultured in DMEM medium (Hyclone) supplemented with 10% FBS (Gibco) and 100 U/mL penicillin/streptomycin (Gibco) at 37 °C with 5% CO_2_.

### Cell immunostaining

The cells were plated on culture slides and fixed in 4% paraformaldehyde for 30 min, washed with PBS, and stained with an anti-α-actinin antibody (Santa Cruz) in immunofluorescence buffer at 4 °C overnight. Then, the sections were washed with PBS and incubated with the secondary antibody (Abways) for 90 min. The nuclei were stained with Hoechst. Images were obtained using a fluorescence inverted microscope (Olympus) and analyzed with ImageJ.

### Cell transfection

The specific shRNA (Sangon Biotech)-targeting METTL3 and DKK2 were designed and cloned into the pLKO.1 vector respectively for the generation of shMETTL3 and shDKK2 to inhibit their expression in NRCMs. The genomic sequence encoding rat METTL3 was cloned into the pLVX vector for the generation of the pLVX-METTL3 vector to overexpress METTL3 in NRCMs. The empty lentivirus vector was set as the negative control. The detailed sequence information of shMETTL3 and shDKK2 are shown in Additional file 1: Table S2. The carrier vectors and helper plasmids were transfected into the HEK293T using Lipofectamine 2000 (Invitrogen) regents according to the manufacturer’s protocol. This process was provided and carried out by GenePharma. The lentivirus was transfected into the NRCMs at a multiplicity of infection (MOI) of 100 for 24 h, and the transfection mixture was replaced with normal complete medium for another 24 h before stimulation with Ang-II. Then, 100 nM mimics (GenePharma) and 100 nM inhibitor (GenePharma) of miR-221/222, as well as the same dose of corresponding negative control (GenePharma), were transfected into NRCMs with help of Lipofectamine 2000 (Invitrogen) kit according to the manufacturer’s instructions for 24 h prior to the addition of Ang-II. Detailed information on the sequence is also shown in Additional file 1: Table S2.

### Real-time PCR analysis

Total RNA was extracted by TRIzol reagent (Invitrogen) from NRCMs and heart tissues following the manufacturer’s instructions. The concentrations of RNA were detected by NanoDrop ND-1000 spectrophotometer (Thermo Scientific). Then, RNA was reverse-transcribed into cDNA using the HiScript QRT Super Mix (Vazyme) and miRNA First-Strand Synthesis Kit (Vazyme) according to the manufacturer’s protocol. The real-time polymerase chain reaction was carried out using IQ SYBR Green Supermix (Bio-Rad) and the Prism 7500 SDS (Applied Biosystems). Finally, the relative expression value of genes was calculated using the 2^−ΔΔCt^ method. U6 was used as the internal control of miR-221/222, and β-actin was used as the internal control for the other genes. The primer sequences (Sangon Biotech) of the genes are shown in Additional file 1: Tables S3 and S4.

### Western blot

Total protein was extracted by Whole-Cell Lysis Assay (KeyGEN BioTECH) from NRCMs and heart tissues, and the concentration was defined by the BCA Protein Assay Kit (KeyGEN BioTECH). Protein extractions were boiled and denatured, separated by 10% SDS–PAGE and then transferred onto PVDF membranes (Millipore). After blocking with 5% skimmed milk powder, the PVDF membranes were incubated overnight at 4 °C with the following rabbit-sourced primary antibodies, including anti-METTL3 antibody (Abcam, ab195352, 1:1000), anti-ANP antibody (Abcam, ab189921, 1:1000), anti-BNP antibody (Abcam, ab239510, 1:1000), anti-DKK2 antibody (Abcam, ab95274, 1:1000), anti-β-catenin antibody (Abcam, ab32572, 1:1000), anti-c-Myc antibody (Abcam, ab32072, 1:1000), and anti-β-actin antibody (ABways, AB0035, 1:5000). Subsequently, the membranes were washed with TBST and incubated with HRP-conjugated secondary antibody (Abways, AB0101, 1:5000; AB0102, 1:2000) at room temperature for 1 h. Finally, bands were detected using ECL (KeyGEN BioTECH) with a chemiluminescence system (Bio-Rad).

### RNA m6A quantification

Total RNA was isolated, and the m6A content was measured using an m6A RNA methylation quantification kit (Epigentek) following the manufacturer’s instruction. Briefly, 200 ng RNA was added to the wells of the assay plate. Then, the capture antibody solution and the detection antibody solution were added to the assay wells according to the manual. The m6A levels were quantified with colorimetry method by reading the absorbance of each well at 450 nm and calculating relative m6A abundance.

### RNA immunoprecipitation (RIP) assay

NRCMs overexpressing METTL3 were UV-irradiated at 254 nm, 400 mJ/cm^2^ (Stratagene Stratalinker), and then lysed with RIP lysis buffer (Absin) at 4 °C through disruptive sonication. Immunoprecipitation of endogenous DGCR8 was performed by anti-DGCR8 antibody (Abcam) or control IgG antibody overnight at 4 °C. After washing, the immunoprecipitated protein–RNA complex was treated with proteinase K. RNAs were extracted by standard phenol/chloroform procedure. Then, the pri-miRNAs were detected with qRT-PCR, and %(IP/Input) and %(IgG/Input) were obtained. Finally, the fold enrichment of m6A level on pri-miRNAs was calculated by %(IP/Input)/%(IgG/Input).

For m6A RIP (meRIP) experiments, RNA extracted from NRCMs overexpressing METTL3 and control NRCMs was treated with deoxyribonuclease I (Sigma). The RNAs were fragmented by sonication for 10 s on the ice-water mixture. Immunoprecipitation was performed using an anti-m6A antibody (Abcam) or control IgG antibody previously bound to magnetic Dynabeads (Life Technologies) in the RIP immunoprecipitation buffer (Millipore) and incubated with DNA-free fragmented RNAs. Beads were then treated with proteinase K (20 mg/mL) for 1.5 h at 42 °C. RNAs were extracted by standard phenol/chloroform procedure. Then, the pri-miRNAs were detected with qRT-PCR, and %(IP/Input) and %(IgG/Input) were obtained. The fold enrichment of pri-miRNAs binding to DGCR8 was calculated by %(IP/Input)/%(IgG/Input).

### Dual-luciferase assay

The target genes of miR-221/222 were predicted by the online webtool TargetScan (http://www.targetscan.org) [18] under default parameters. On the basis of the predicted intersection of target genes in three species of human, rat, and mouse, Gene Ontology (GO) and Kyoto Encyclopedia of Genes and Genomes (KEGG) analyses were performed with another bioinformatic online tool, Enrichr (https://maayanlab.cloud/Enrichr/) [19].

The dual-luciferase assay was applied for validating the predicted connection between miR-221/222 and DKK2. Briefly, the psiCHECK2 plasmids (Promega) containing wild-type DKK2 (DKK2-wt) or DKK2 mutated (DKK2-mut) at the putative miR-221/222 binding sites were constructed. The psiCHECK2-DKK2-wt and psiCHECK2-DKK2-mut plasmids with either miR-221/222 mimic or negative control were delivered into HEK293T cells by Lipofectamine 2000 (Invitrogen) for 48 h. Subsequently, the HEK293T cell lysate was added to Renilla luciferase or firefly luciferase, and the luminescence was determined by SpectraMax M5 (Molecular Devices). The firefly luciferase was used as an internal control.

### Statistical analysis

All data were analyzed by SPSS 22.0 software, and expressed in the form of mean ± standard deviation. Statistical comparisons were performed using unpaired two-tailed *t*-test when two groups were compared. Comparisons of data among multiple groups were carried out by one-way analysis of variance (ANOVA). *P* < 0.05 was regarded as statistically significant.

## Results

### METTL3 and m6A were upregulated in Ang-II-induced cardiac hypertrophy

**Fig. 1:**
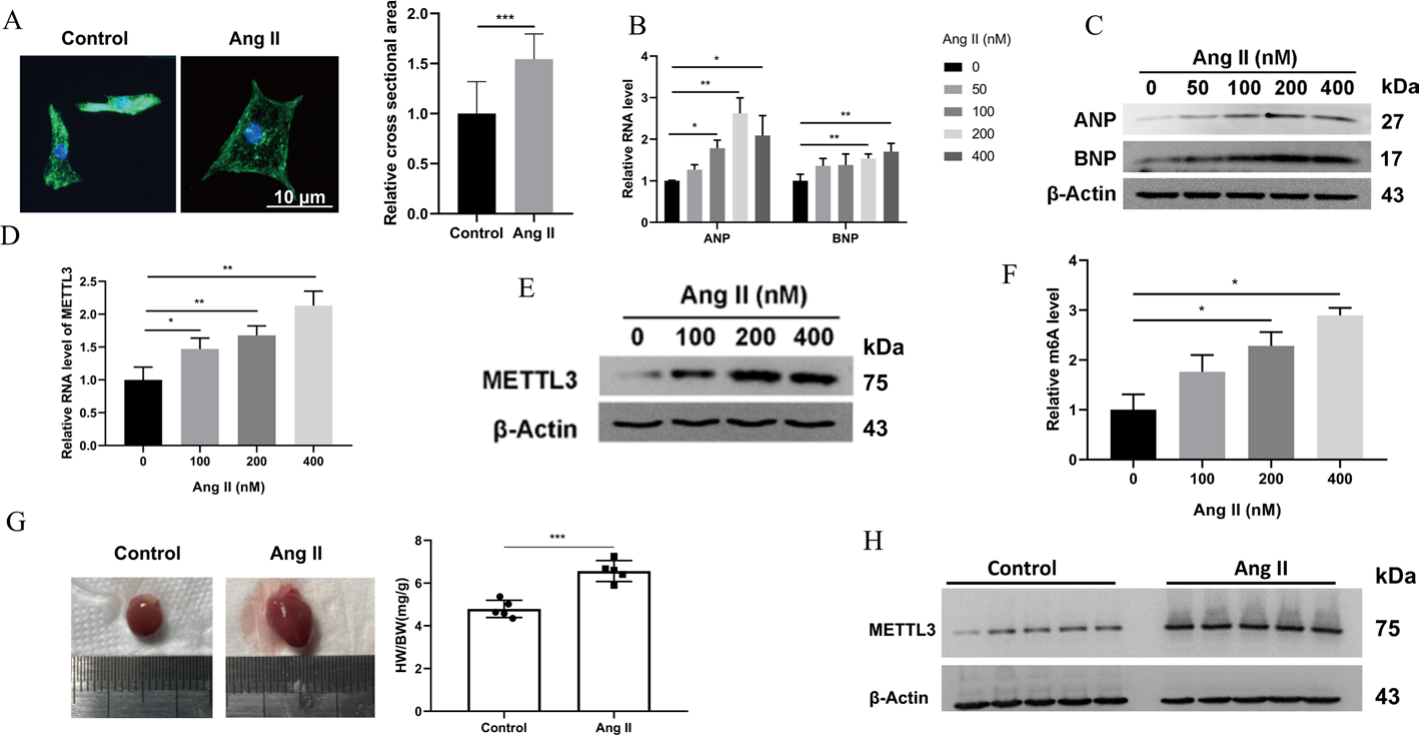
Expression of METTL3 was upregulated in hypertrophic myocardium induced by Ang-II. **A** NRCMs were treated with Ang-II to induce hypertrophy. Representative immunohistochemistry images of NRCMs stained with α-actinin in green. The nucleus is stained in blue. Scale bars, 10 μm. The cell surface area was evaluated. *n* = 3. **B** qRT-PCR detected the expression of hypertrophic biomarkers ANP and BNP in NRCMs stimulated with Ang-II. *n* = 3. **C** Western blot detected the expression of hypertrophic biomarkers ANP and BNP in NRCMs stimulated with Ang-II. *n* = 3. **D** qRT-PCR detected METTL3 expression in Ang-II-induced myocardial hypertrophy in vitro. *n* = 3. **E** Western blot detected the METTL3 expression in Ang-II-induced myocardial hypertrophy in vitro. *n* = 3. **F** Assay detected m6A modification of total RNA from Ang-II-induced NRCMs. *n* = 3. **G** Representative heart gross morphology. The HW:BW ratio was calculated by dividing heart weight by body weight. *n* = 5. **H** Western blot detected METTL3 expression in the myocardium of Ang-II treated mice. *n* = 5. *P < 0.05, **P < 0.01, ***P < 0.001

In this study, Ang-II could significantly stimulate cardiac hypertrophy in NRCMs by increasing the cross-sectional cell area (Fig. 1A) and level of fetal genes, including ANP and BNP (Fig. 1B, C). During the process, METTL3 expression was significantly upregulated (Fig. 1D, E). Considering the vital role of METTL3 in m6A modification, the m6A level was also evaluated and found to be elevated (Fig. 1F). The in vivo model was constructed by infusion of Ang-II in C57BL/6J mice for 3 weeks, after which the myocardium showed significant hypertrophy manifested as significant enlargement of the heart and an increase in the HW:BW ratio (Fig. 1G). Undoubtedly, METTL3 expression was also upregulated in the hypertrophic myocardium (Fig. 1H).

### Knockdown of METTL3 suppressed myocardial hypertrophy in vitro

**Fig. 2:**
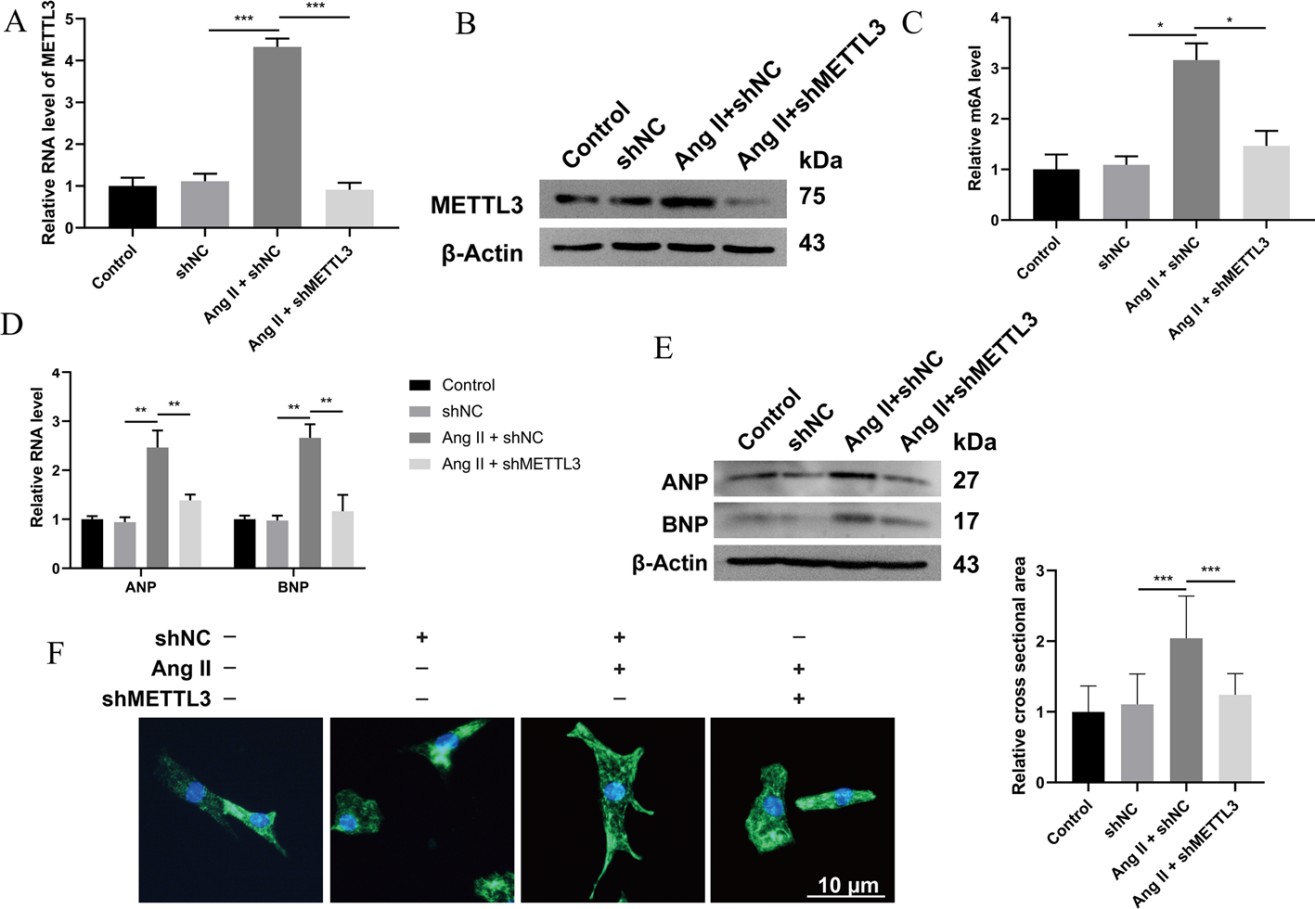
Knockdown METTL3 expression inhibits Ang-II-induced cardiomyocyte hypertrophy. **A** qRT-PCR detected the expression of METTL3 in Ang-II-induced NRCMs transfected with shMETTL3. *n* = 3. **B** Western blot detected the expression of METTL3 in Ang-II-induced NRCMs transfected with shMETTL3. *n* = 3. **C** Assay detected m6A modification of total RNA in Ang-II-induced NRCMs transfected with shMETTL3. *n* = 3. **D** qRT-PCR detected the expression of biomarkers ANP and BNP in Ang-II-induced NRCMs transfected with shMETTL3. *n* = 3. **E** Western blot detected the expression of biomarkers ANP and BNP in Ang-II-induced NRCMs transfected with shMETTL3. *n* = 3. **F** NRCMs were transfected with shMETTL3, followed by Ang-II stimulation. Representative immunohistochemistry images of NRCMs stained with α-actinin in green. The nucleus is stained in blue. Scale bars represent 10 μm. The cell surface area was evaluated. *n* = 3. *P < 0.05, **P < 0.01, ***P < 0.001

To date, whether METTL3 regulates Ang-II-induced hypertrophy and whether it regulates through m6A are still unknown. To clarify its role in this disease, NRCMs were transfected with shMETTL3 or pLVX-METTL3 to inhibit or activate its expression at the cellular level. METTL3 expression was effectively downregulated (Additional file 1: Fig. S1A, B) or upregulated, respectively (Additional file 1: Fig. S1C, D), by the corresponding vectors. Furthermore, Ang-II-induced upregulation of METTL3 (Fig. 2A, B) and increased m6A level (Fig. 2C) were reversed by shMETTL3. At the same time, the upregulation of ANP and BNP (Fig. 2D, E) and the increase in cell cross-sectional area (Fig. 2F) in Ang-II-stimulated NRCMs were also hindered by METTL3 knockdown. These results suggested that METTL3 could mediate Ang-II-induced myocardial hypertrophy and the increased m6A modification.

### METTL3 promotes the processing of miR-221/222 by DGCR8 in an m6A-dependent manner

**Fig. 3:**
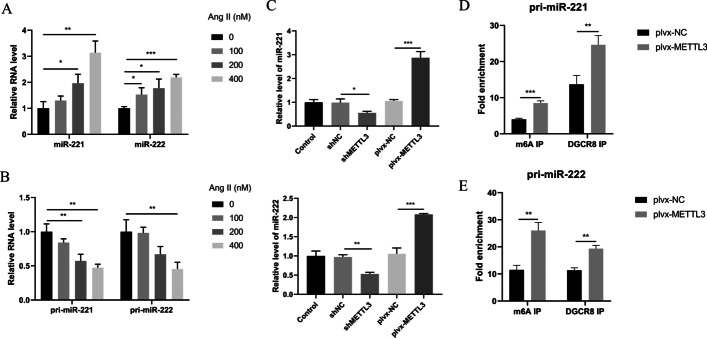
Maturation of miR-221/222 was promoted by METTL3 in an m6A-dependent manner. **A** qRT-PCR detected the expression of miR-221 and miR-222 in Ang-II-induced NRCMs. *n* = 3. **B** qRT-PCR detected the expression of pri-miR-221 and pri-miR-222 in Ang-II-induced NRCMs. *n* = 3. **C** METTL3 was overexpressed and inhibited respectively after transfecting plvx-METTL3 or shMETTL3 into NRCMs. qRT-PCR detected the expression of miR-221 and miR-222. *n* = 3. **D** Detection of pri-miR-221 binding to DGCR8 or m6A modification by immunoprecipitation experiments in control and METTL3 overexpression NRCMs. *n* = 3. **E** Detection of pri-miR-222 binding to DGCR8 or m6A modification by immunoprecipitation experiments in control and METTL3 overexpression NRCMs. *n* = 3. *P < 0.05, **P < 0.01, ***P < 0.001

It was reported that miR-221/222 was associated with cardiac hypertrophy and promoted by METTL3 in an m6A-dependent manner. We further assessed the connection between miR-221/222 with METTL3 in NRCMs. In the in vitro model of Ang-II-induced cardiac hypertrophy, miR-221 and miR-222 expression were significantly upregulated (Fig. 3A), while their pri-miRNA levels were downregulated (Fig. 3B). Overexpression of METTL3 in NRCMs was sufficient to promote miR-221/222 expression and downregulation of METTL3 resulting in miR-221/222 inhibition (Fig. 3C), indicating that miR-221/222 is positively regulated by METTL3.

To verify whether m6A mediated the regulation of METTL3 on these two miRNAs, m6A sites among pri-miR-221/222 were firstly evaluated (Additional file 1: Fig. S2) according to the rule that the m6A modification is located in the specific conserved motif DRACH (D = A, G or U; R = A or G; H = A, C or U) [20]. To further test whether METTL3 mediates the increase of miR-221/222 by promoting DGCR8 binding to m6A modified pri-miR-221/222 in cardiomyocytes, immunoprecipitation assay was performed using anti-DGCR8 and anti-m6A antibodies. The results showed that the levels of pri-miR-221/222 binding with DGCR8 increased when METTL3 was overexpressed (Fig. 3D, E). Besides, METTL3 overexpression could significantly increase the modification level of m6A on pri-miR-221/222 (Fig. 3D, E). Taken together, the data suggest that METTL3 could promote the maturation process of pri-miR-221/222 by DGCR8 in an m6A-dependent manner.

### miR-221/222 act against the effects of METTL3 knockdown on myocardial hypertrophy

**Fig. 4:**
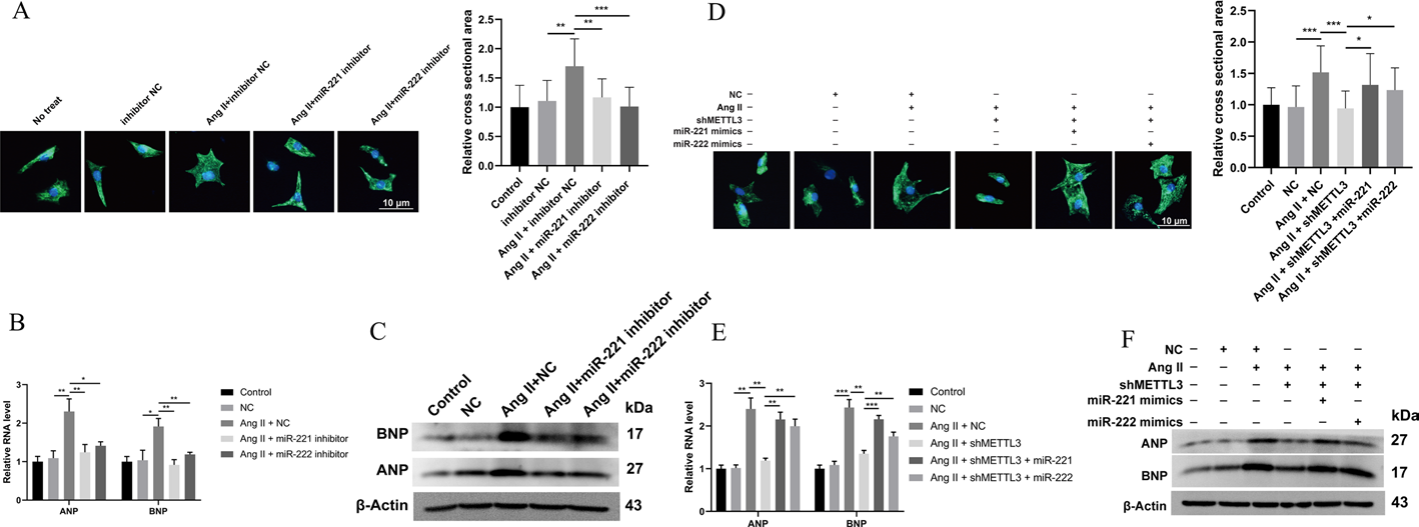
miR-221/222 mediated the regulation of METTL3 on Ang-II-induced cardiomyocyte hypertrophy. **A** miR-221 and miR-222 in NRCMs were inhibited by the specific inhibitors during Ang-II-induced hypertrophy. Representative immunohistochemistry images of NRCMs stained with α-actinin in green. The nucleus stained in blue. Scale bars, 10 μm. Cell surface area was evaluated. *n* = 3. **B** qRT-PCR detected the expression of hypertrophic biomarkers ANP and BNP in Ang-II-induced NRCMs transfected with inhibitors of miR-221/222. *n* = 3. **C** Western blot detected the expression of hypertrophic biomarkers ANP and BNP in Ang-II-induced NRCMs transfected with inhibitors of miR-221/222. *n* = 3. **D** miR-221 mimics and miR-222 mimics were transduced into METTL3-knockdown NRCMs and stimulated with Ang-II. Representative immunohistochemistry images of NRCMs stained with α-actinin in green. The nucleus is stained in blue. Scale bars, 10 μm. Cell surface area was evaluated. *n* = 3. **E** qRT-PCR detected the expression of biomarkers ANP and BNP in Ang-II-induced NRCMs transfected with shMETTL3 and miR-221/222 mimics. *n* = 3. **F** Western blot detected the expression of biomarkers ANP and BNP in Ang-II-induced NRCMs transfected with shMETTL3 and miR-221/222 mimics. *n* = 3. *P < 0.05, **P < 0.01, ***P < 0.001

To investigate the action of miR-221/222 on Ang-II-induced myocardial hypertrophy, miR-221/222 inhibitors and mimics were transfected into cells, with or without inhibition of METTL3 expression. Ang-II promoted cross-sectional area increase of NRCMs, and miR-221/222 inhibitors could effectively counteract that pathological change (Fig. 4A). The same results could be observed in terms of the expression of ANP and BNP (Fig. 4B, C). Moreover, inhibition of METTL3 counteracted the increase in cell area (Fig. 4D) and the upregulation of the hypertrophic biomarkers (Fig. 4E, F) induced by Ang II. However, miR-221/222 mimics could reverse the therapeutic effect of METTL3 knockdown, resulting again in increased cell cross-sectional area and upregulation of ANP and BNP (Fig. 4D–F). Thus, METTL3 could mediate the Ang-II-induced myocardial hypertrophy by upregulating miR-221/222.

### Wnt/β-catenin pathway was regulated by METTL3–miR-221/222 in NRCMs

**Fig. 5:**
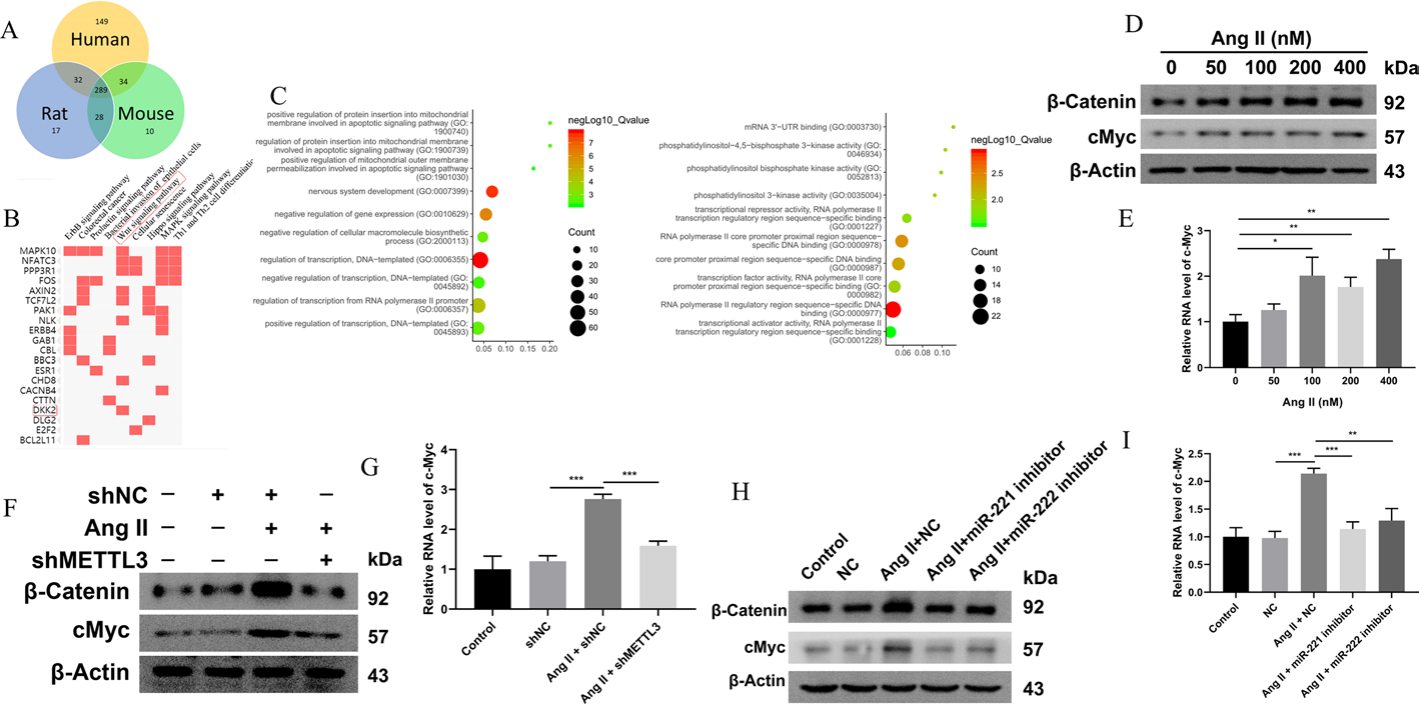
Wnt/β‐catenin was downstream regulated by METTL3 and miR-221/222. **A** Venn diagram indicating the targets of miR-221/222 in human, rat, and mouse. **B** KEGG analysis of the 289 intersection target genes of three species. **C** GO analysis of the 289 intersection target genes of three species. **D** Western blot detected the expression of β‐catenin and c-Myc in NRCMs stimulated with Ang-II. *n* = 3. **E** qRT-PCR detected the expression of c-Myc in NRCMs stimulated with Ang-II. *n* = 3. **F** Western blot detected the expression of β‐catenin and c-Myc in Ang-II-induced NRCMs with METTL3 knockdown. *n* = 3. **G** qRT-PCR detected the expression of c-Myc in Ang-II-induced NRCMs with METTL3 knockdown. *n* = 3. **H** Western blot detected the expression of β‐catenin and c-Myc in Ang-II-induced NRCMs with miR-221/222 inhibition. *n* = 3. **I** qRT-PCR detected the expression of c-Myc in Ang-II-induced NRCMs with miR-221/222 inhibition. *n* = 3. *P < 0.05, **P < 0.01, ***P < 0.001

It is well known that miRNAs usually target and inhibit mRNA expression in biology. Therefore, the target genes of miR-221/222 were predicted by the online tool TargetScan. As shown in the results, there were hundreds of genes predicted in human, rat, and mouse species, and 289 genes were at the intersection of these three groups (Fig. 5A). GO and KEGG analysis indicated that these 289 genes were enriched in ErbB signaling pathway, Wnt signaling pathway, etc. (Fig. 5B, C).

As activation of the Wnt/β-catenin pathway is vital in cardiac hypertrophy [21], it was selected as a potential mediator of METTL3–miR-221/222 to convey the hypertrophic effect. The status of the signaling pathway was evaluated by detecting β-catenin and downstream target c-Myc. During Ang-II-induced cardiac hypertrophy, β-catenin expression was significantly upregulated (Fig. 5D). Besides, c-Myc, the target of Wnt signaling, was also upregulated (Fig. 5D, E), indicating the activation of the Wnt/β-catenin signaling pathway. The increase of β-catenin and c-Myc in NRCMs stimulated with Ang-II was conversely downregulated by inhibiting METTL3 expression (Fig. 5F, G), as well as by inhibiting miR-221/222 (Fig. 5H, I), suggesting that METTL3 and miR-221/222 could regulate its activity respectively. Finally, to further confirm the signaling as the downstream target of the METTL3–miR-221/222 axis, the Wnt pathway was assessed in NRCMs with METTL3 inhibition and miR-221/222 overexpression. The results showed that the downregulated β-catenin and c-Myc expression by METTL3 knockdown was re-upregulated by miR-221/222 mimics (Additional file 1: Fig. S3A, B). Altogether, METTL3-induced miR-221/222 expression mainly promoted pathological cardiac hypertrophy in Ang-II via activating Wnt/β-catenin signaling.

### DKK2 was the direct target of miR-221/222 in regulating Wnt signaling

**Fig. 6:**
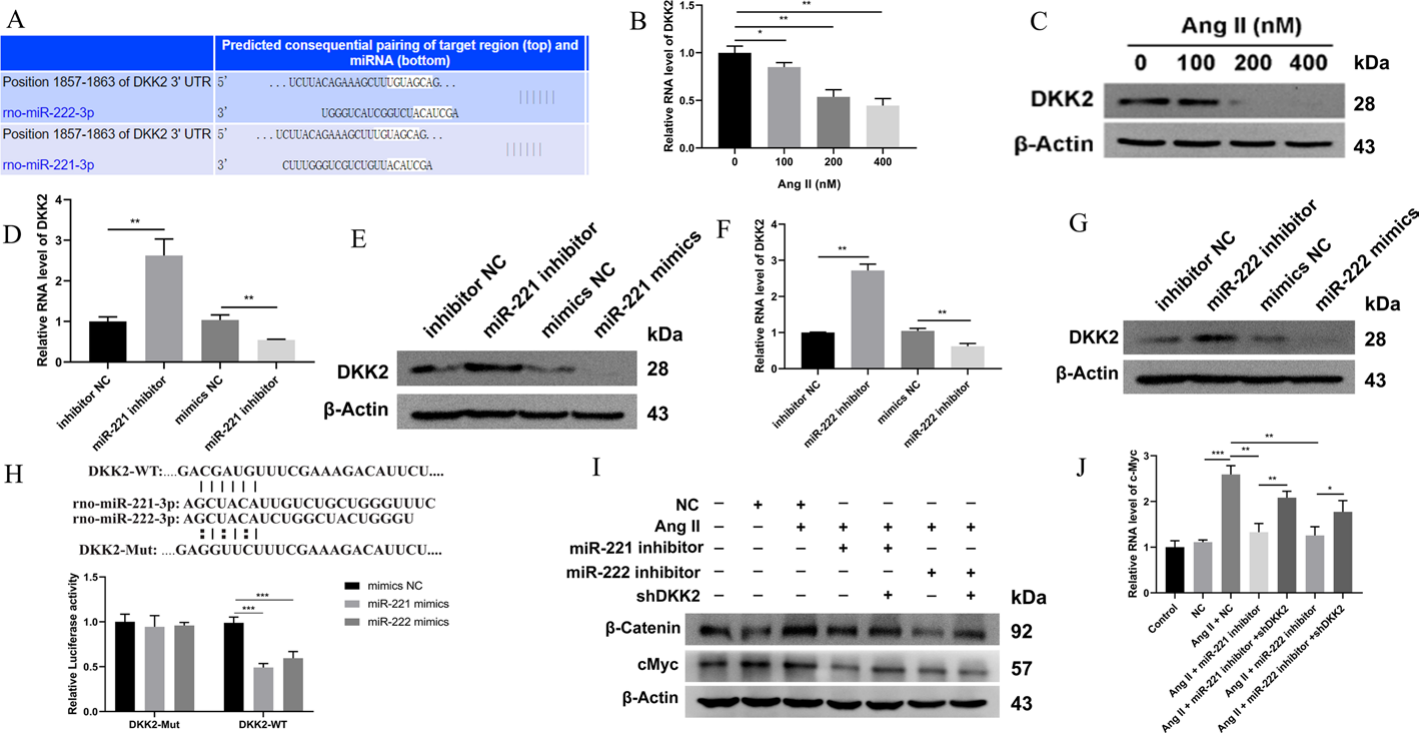
DKK2 was the direct target of miR-221/222 in regulating Wnt signaling in NRCMs. **A** The miR-221/222 binding site in the 3′-UTR of DKK2 predicted by TargetScan database. **B** qRT-PCR detected the expression of DKK2 in NRCMs stimulated with Ang-II. *n* = 3. **C** Western blot detected the expression of DKK2 in NRCMs stimulated with Ang-II. *n* = 3. **D** qRT-PCR detected the expression of DKK2 in NRCMs transduced with miR-221 mimics and inhibitors. *n* = 3. **E** Western blot detected the expression of DKK2 in NRCMs transduced with miR-221 mimics and inhibitors. *n* = 3. **F** qRT-PCR detected the expression of DKK2 in NRCMs transduced with miR-222 mimics and inhibitors. *n* = 3. **G** Western blot detected the expression of DKK2 in NRCMs transduced with miR-222 mimics and inhibitors. *n* = 3. **H** Predictive miR-221/222 binding sites in the 3′-UTR of DKK2 mRNA. Dual-luciferase reporter assays demonstrated that DKK2 was a direct target of miR-221/222. **I** Western blot detected the expression of β‐catenin and c-Myc in Ang-II-induced NRCMs with miR-221/222 inhibition and DKK2 knockdown. *n* = 3. **J** qRT-PCR detected the expression of c-Myc in Ang-II-induced NRCMs with miR-221/222 inhibition and DKK2 knockdown. *n* = 3. *P < 0.05, **P < 0.01, ***P < 0.001

Several genes, including MAPK10, NFATC3, and DKK2, were predicted to be downstream targets of miR-221/222 involved in the Wnt/β-catenin signaling pathway (Fig. 5A). DKK2 is an inhibitor of Wnt/β-catenin in various diseases [22, 23]. However, whether DKK2 is a participator in cardiac hypertrophy is still unclear. According to TargetScan, position 1857–1863 in the 3′-untranslated region (3′-UTR) of DKK2 was potentially targeted by miR-221/222 (Fig. 6A), which suggested that these two miRNAs may inhibit DKK2 expression. As expected, DKK2 expression was downregulated in Ang-II-stimulated NRCMs (Fig. 6B, C). Both miR-221 mimics (Fig. 6D, E) and miR-222 mimics (Fig. 6F, G) could inhibit DKK2 expression, while their inhibitor (Fig. 6D–G) promoted the expression of DKK2, indicating that miR-221 and miR-222 negatively regulated DKK2 in CMs.

To further evaluate the action of miR-221/222 on DKK2, luciferase report assay was performed by constructing the mutated sequence at the above position. The results showed that miR-221/222 could negatively regulate the luciferase activity in the DKK2-WT group but not in the DKK2-MUT group (Fig. 6H). Besides, miR-221/222 mimics could cause the luciferase activity in the DKK2-WT group to be lower than that in the DKK2-MUT group, while mimics NC did not have the same effect (Fig. 6H). Combined, these results suggest that miR-221/222 could directly inhibit DKK2 expression in CMs.

Subsequently, DKK2 was inhibited to explore its function in miR-221/222 regulating cardiac hypertrophy. As shown, the expression of DKK2 was downregulated effectively by the shDKK2s (Additional file 1: Fig. S4A, B). When DKK2 in NRCMs was knocked down, the negative effect of miR-221/222 inhibitors on Ang-II-induced Wnt activation was abolished (Fig. 6I, J). Besides, the expression of the biomarkers of hypertrophy including ANP and BNP downregulated by miR-221/222 inhibitors was also restored after DKK2 knockdown (Additional file 1: Fig. S5A–B). Therefore, DKK2 was an important factor in mediating the function of miR-221/222 in the Wnt/β-catenin signaling pathway and hypertrophy of the myocardium.

### AAV9-mediated METTL3 inhibition alleviated cardiac remodeling of mice under Ang-II stimulation

**Fig. 7:**
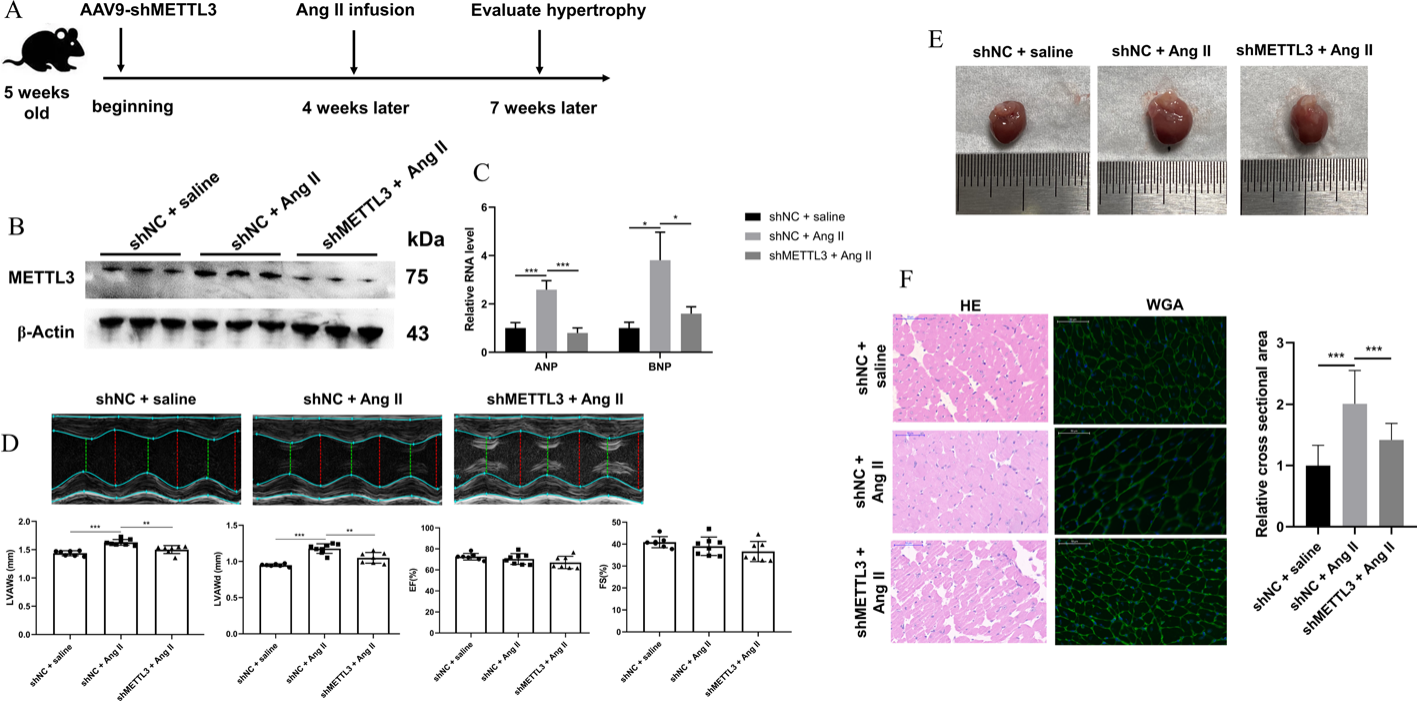
AAV9-delivery system carrying shMETTL3 alleviates the degree of hypertrophy in mice. **A** Workflow of animal research. **B** Western blot detected the expression of METTL3 in AAV9-shMETTL3 transfected with mice infused with Ang-II. *n* = 3. **C** qRT-PCR detected the expression of biomarkers, including ANP and BNP, in the heart of the three groups. *n* = 4. **D** Cardiac color Doppler ultrasound evaluated the indexes of cardiac structure, including LVAWs, LVAWd, EF, and FS. *n* = 7 in shNC group, *n* = 8 in Ang-II + shNC group, *n* = 7 in Ang-II + shMETTL3 group. **E** Representative heart gross morphology from the mice among three groups. *n* = 5. **F** Histological sections stained with H&E and WGA from mice of the three groups. *n* = 5. Scale bars, 50 μm. *P < 0.05, **P < 0.01, ***P < 0.001

To determine the therapeutic effect of METTL3 knockdown in vivo, AAV9 was utilized to deliver shMETTL3 to the heart of mice via caudal vein 4 weeks before inducement of myocardial hypertrophy with Ang-II (Fig. 7A). As expected, Ang-II significantly promoted METTL3 expression in the heart, and AAV9-shMETTL3 significantly decreased expression of METTL3 in the remodeling heart (Fig. 7B), suggesting that the AAV9 vector successfully delivered shMETTL3 into the diseased cardiac system. Besides, miR-221/222 and β-catenin were upregulated in Ang-II-induced hypertrophic heart and reversely downregulated by METTL3 knockdown (Additional file 1: Fig. S6A, B).

We further evaluated the action of METTL3 knockdown on the cardiac hypertrophy in vivo. As shown, the hypertrophic biomarkers, including ANP and BNP, were upregulated in the hypertrophic hearts stimulated by Ang-II and also reversely downregulated after knocking down METTL3 despite Ang-II stimulation (Fig. 7C), consistent with the in vitro results. Moreover, echocardiography indicated that Ang-II-caused increase of LVAW in systole and diastole was mitigated by METTL3 knockdown, while the values of EF and FS were not imbalanced after Ang-II infusion or combined inhibition of METTL3 (Fig. 7D). The size of the heart among the three groups was evaluated, and the results showed that METTL3 knockdown reduced the heart enlargement induced by Ang-II (Fig. 7E). Finally, H&E and WGA staining showed that treatment with anti-METTL3 AAV9 vector significantly reduced the cross-sectional area of cardiomyocytes in hypertrophic myocardium (Fig. 7F). Overall, METTL3 inhibition treatment carried out by AAV9-delivery system had excellent performance in alleviating cardiac hypertrophy.

## Discussion

*N*^6^-methyladenosine (m6A) is regarded as the most prevalent internal RNA modification within eukaryotic mRNAs, with METTL3, METTL14, and WTAP being core methyltransferases and FTO and ALKBH5 the core demethylases [24]. Over the past years, there have been multiple studies regarding the role of m6A in regulating various cardiovascular biological processes such as myocardial ischemia/hypoxia [25] or ischemia/reperfusion injury [26], and myocardial proliferation [27]. Recently, METTL3 was reported to be upregulated in Ang-II-induced myocardial hypertrophy [9]. However, whether METTL3-mediated m6A modification participates in this pathological remodeling remains unclear.

In the present study, METTL3 expression was discovered to be elevated in Ang-II-induced hypertrophic myocardium in vivo and in vitro, same as previously reported by Lu et al. [9]. This was also similar to another previous study in which the expression of METTL3 was higher in clinical failing heart samples from patients with left ventricular hypertrophy than in samples from healthy subjects without heart failure [28]. We further found that METTL3 knockdown significantly alleviated Ang-II stimulated cardiomyocyte remodeling, suggesting that METTL3 is involved in the formation of Ang-II-induced myocardial hypertrophy. Recently, several studies indicated a double-edged sword function of METTL3-m6A in regulating cardiac hypertrophy induced by different stimulating factors. As revealed by Dorn et al. [29], METTL3 was a key factor driving the m6A in cardiac hypertrophy, and knockdown/knockout of METTL3 could relieve the hypertrophy under serum or pressure overload stimulation. However, this contradicts another study that proposed that METTL3-dependent m6A was a vital disease resistance factor in transverse aortic constriction or phenylephrine-induced cardiac remodeling [30]. In fact, similar contradictory results regarding the role of METTL3 have been observed in other cardiovascular diseases. For example, a recent study discovered that METTL3 knockdown could promote CM proliferation [31], while in another METTL3 was proven to activate the proliferation of CMs [32], which highly suggested that the biological function of m6A modification is extremely complex in the cardiac system. Our results were based on Ang-II stimulation, which mainly exerts its function through the angiotensin type 1 receptor (AT1R), while phenylephrine compound predominantly acts as α(1) adrenergic receptor agonist [33, 34]. AT1R is distributed among multiple cardiovascular tissues, including myocardial and vascular smooth muscle [35]. Interestingly, the levels of METTL3 in smooth muscle cells of the aortic were also significantly upregulated in Ang-II-induced mouse abdominal aortic aneurysm model [36]. Therefore, we conclude that the role of METTL3/m6A in myocardial hypertrophy caused by different stimulating factors may depend on the function of the corresponding receptor with its downstream signaling. It is necessary to further clarify how METTL3 participates in the regulation of myocardial hypertrophy response to Ang-II stimulation.

During Ang-II-promoted METTL3 expression in cardiomyocytes, the level of m6A modification in the RNA was also increased, and conversely reduced in METTL3-silenced cells. We thus speculated that the METTL3-mediated hypertrophy effect by Ang-II occurred mainly through its m6A modification function. So far, most types of RNA, including mRNA, miRNA, and lncRNA, have been shown to be modified and manipulated by m6A [37]. Lu et al. [9] and Dorn et al. [29] both identified the pro-hypertrophic family mitogen-activated protein kinases (MAPKs) as the downstream target modified by METTL3/m6A during the hypertrophy process, but could not rule out a role of noncoding RNA. In fact, m6A involvement in miRNA maturation is an important factor in the pathogenesis of cardiovascular diseases [36, 38] and other diseases including lung fibrosis [39]. Multiple miRNAs, including miR-221/222 cluster, were proven to have their primary forms’ maturation accelerated by m6A modification [14]. In an Ang-II-constructed mouse model of cardiac hypertrophy, miR-221/222 expression was simultaneously increased in cardiac tissues. However, the adrenergic system stimulated by isoprenaline to induce the hypertrophy model did not display a substantial increase in miR-221/222 expression [12]. This also reflects the hypertrophic effects mediated by different receptors depending on different mechanisms. To clarify whether miR-221/222 was directly regulated by METTL3 in Ang-II-induced cardiac hypertrophy, we evaluated the expression of these two miRNAs and confirmed that miR-221/222 was upregulated in Ang-II-stimulated cardiomyocytes. Specific inhibitors targeting miR-221/222 could retard the progression of hypertrophy induced by Ang-II, similar to the previous report in which miR-221 could drive spontaneous cardiac hypertrophy in vitro [13]. In addition, miR-221/222 was positively regulated by METTL3 through m6A modification on the pri-miR-221/222 sequences, and their overexpression could resist the protective behavior of METTL3 knockdown, pointing out the significance of the METTL3–miR-221/222 axis in pathological remodeling.

As is well known, miRNAs are a type of small noncoding RNA that targets mRNAs by binding with the 3′-UTR, resulting in mRNA expression suppression [40]. After bioinformatics analysis, hundreds of genes were obtained from the TargetScan database that were potentially targeted by miR-221/222. The Wnt/β-catenin pathway, a recognized promoter of hypertrophy, was significantly enriched as the downstream of the METTL3–miR-221/222 axis by KEGG analysis on these genes. The Wnt/β-catenin signaling is an evolutionarily conserved regulator in development and homeostasis, and its activation is characterized by increased accumulation of β-catenin in the cytoplasm and subsequent migration into the nucleus, thus promoting target gene expression, including c-Myc [41]. In our study, Wnt/β-catenin was activated by Ang-II, repressed by METTL3 knockdown or miR-221/222 inhibitors, and reactivated by miR-221/222 mimics despite inhibition of METTL3 expression. Interestingly, upregulated METTL3 was reported to promote β-catenin and c‐Myc expression, and mainly affect the biological progression of hepatoblastoma through the Wnt/β‐catenin pathway [42]. This result, combined with ours, indicated that METTL3–miR-221/222 signaling and Wnt signaling were closely connected in terms of molecular function. In our bioinformatics analysis, DKK2, a gene enriched in the Wnt/β‐catenin pathway, was identified as a potential direct target of miR-221/222, and further demonstrated by detecting its expression in cells treated with Ang-II, miRNA mimics, and inhibitors, as well as by luciferase activity. DKK2 belongs to the dickkopf (DKK) family, and is a secreted antagonist in Wnt/β-catenin signaling by binding to the lipoprotein receptor-related protein 5/6 (LRP5/6) component [43]. In cardiac fibroblasts, DKK2 was reported to suppress Wnt signaling, thus inhibiting cell proliferation and migration [44]. In cardiomyocytes, DKK2 was also proven by our results to be the negative modulator of Wnt/β‐catenin signaling, and functioned as the intermediary in miR-221/222 regulating Wnt/β‐catenin, which were revealed in previous work of breast cancer [23]. Several other members of the DKK family, including DKK3 and DKK1, have been successively proven to be antimyocardial hypertrophy factors, and the Wnt pathway has been shown to be the downstream core [45, 46]. Therefore, the METTL3–miR-221/222-DKK2 signaling pathway was vital in Ang-II-induced activation of Wnt/β‐catenin and the phenotype of cardiomyocytes.

Recombinant AAVs have emerged as a highly promising vector for clinical gene therapy [47]. AAV9 was applied in our research to deliver the shRNA targeting METTL3 to the heart. Since METTL3 knockout in the heart alone has been found to reduce cardiomyocyte cross-sectional area under aging without Ang-II stimulation [29], we evaluated only the effect of METTL3 knockdown on Ang-II-induced myocardial hypertrophy. The results showed that the increased wall thickness, cross-sectional area of cardiomyocytes, and hypertrophic biomarkers in the remodeling hearts of the mice decreased significantly after cardiac knockdown of METTL3. This should not benefit from the improvement of blood pressure, because a previous report has shown that caudal vein administration of AAV9-shMETTL3 inhibiting METTL3 expression in the artery did not influence hypertension induced by Ang-II [36]. Besides, the cardiac function index including EF and FS did not fluctuate after METTL3 knockdown. However, according to Dorn et al. [29], although the hypertrophy in cardiomyocyte-specific METTL3 knockout (METTL3-cKO) mice showed remission in response to pressure overload, they also displayed maladaptive eccentric remodeling with signs of heart failure. Therefore, the AAV9 vector carrying shMETTL3 has unique advantages in improving cardiac hypertrophy.

## Conclusions

**Fig. 8:**
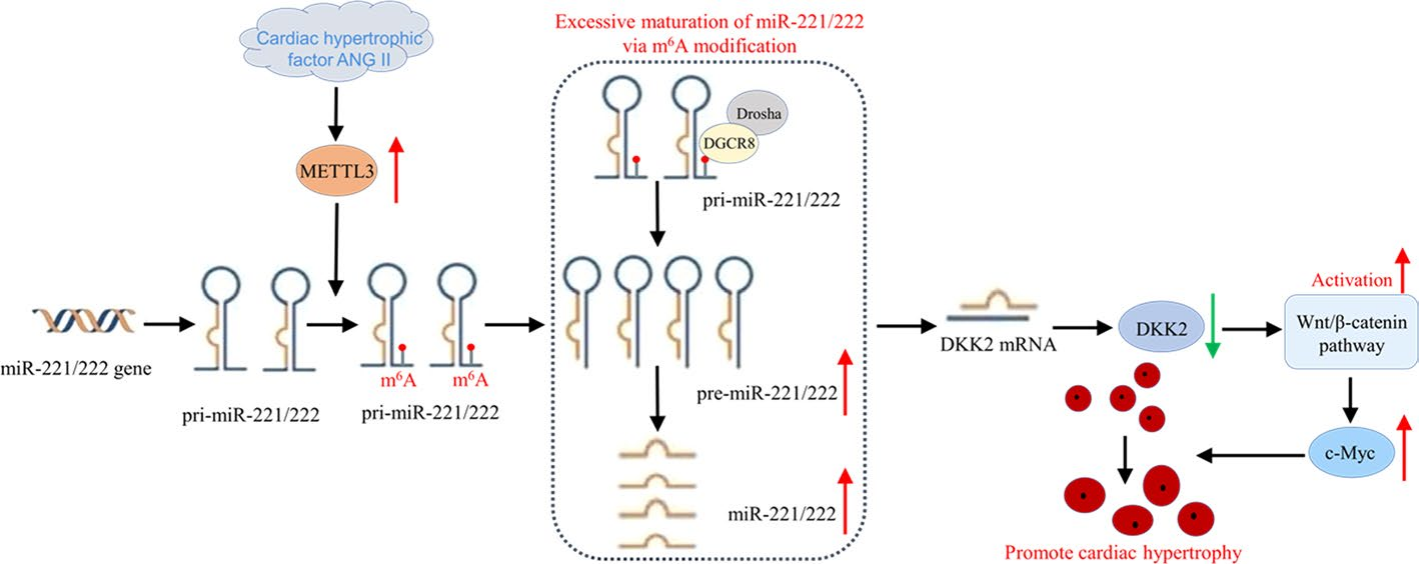
Graphical abstract of the whole study. METTL3 in CMs was upregulated in response to Ang-II, promoting m6A modification on pri-miR-221/222, thus leading to increased miR-221/222 expression. Subsequently, DKK2 was inhibited and Wnt/β‐catenin/c-Myc pathway was activated, finally promoting myocardial hypertrophy

The current study demonstrates a vital regulatory role of METTL3 in Ang-II-induced cardiac hypertrophy. In particular, we highlight the importance of m6A-miRNA mechanism in which METTL3 positively promotes miR-221/222 maturation via modifying pri-miR-221/222 in an m6A-dependent manner, subsequently activating the Wnt/β-catenin pathway by inhibiting DKK2 (Fig. 8). AAV9 delivery system targeting METTL3 is a promising method in the treatment of cardiac remodeling.

## Supplementary Information

**Additional file 1: Figure S1.** Identification of knockdown and overexpression efficiency regarding METTL3. NRCMs were transfected with lentivirus vector carrying three different shRNA sequences targeting METTL3. qRT-PCR (A) and Western blot (B) detected the expression of METTL3. n = 3. NRCMs were transfected with plvx-METTL3 vector to overexpress METTL3. qRT-PCR (C) and Western blot (D) detected the expression of METTL3. n = 3. **P < 0.01, ***P < 0.001. **Figure S2.** The predicted m6A site in sequences of pri-miR-221 and pri-miR-222. Sequences marked in blue and red combined represent the pre-miR-221 (A) and pre-miR-222 (B), while marked in red only as the mature miR-221 (A) and miR-222 (B). The m6A site of pri-miR-221 (A) and pri-miR-222 (B) was marked in green. **Figure S3.** Evaluate the Wnt/β-catenin in NRCMs transfected with shMETTL3 and miR-221/222 mimics. Western blot detected the expression of β‐catenin and c-Myc (A) in Ang-II induced NRCMs transfected with shMETTL3 and miR-221/222 mimics. n = 3. qRT-PCR detected the expression of c-Myc (B) and DKK2 (C) in Ang-II induced NRCMs transfected with shMETTL3 and miR-221/222 mimics. n = 3. *P < 0.05, **P < 0.01. **Figure S4.** Identification of knockdown efficiency regarding DKK2. NRCMs were transfected with lentivirus vector carrying three different shRNA sequences targeting DKK2. qRT-PCR (A) and Western blot (B) detected the expression of DKK2. n = 3. ***P < 0.001. **Figure S5.** Evaluate the effect of DKK2 knockdown on hypertrophy remission induced by miR-221/222 inhibition. qRT-PCR detected the expression of ANP (A) and BNP (B) in Ang-II induced NRCMs with miR-221/222 inhibition and DKK2 knockdown. n = 3. *P < 0.05, **P < 0.01, ***P < 0.001. **Figure S6.** Evaluate the regulation of METTL3 knockdown on miR-221/222 and Wnt/β-catenin signaling in mouse model. qRT-PCR detected the expression of miR-221 (A) and miR-222 (A) in the three groups. n = 3. Western blot detected the expression of β-catenin (B) in the three groups. n = 3. *P < 0.05, **P < 0.01. **Table S1.** Sequences of knockdown in Mouse. **Table S2.** Sequences of knockdown or overexpression in Rat. **Table S3.** Sequences of Primer for Rat. Table S4. Sequences of Primer for mouse.

## Data Availability

The data used to support the findings of this study are available from the corresponding author or first author upon reasonable request.

## Abbreviations

Ang-II: Angiotensin II
AAV9: Adeno-associated virus 9
ANP: Atrial natriuretic peptide
BNP: Brain natriuretic peptide
βMHC: β-Myosin heavy chain
RAS: Renin–angiotensin system
SD: Sprague-Dawley
shRNA: Short-hairpin RNA
LVAW: Left ventricular anterior wall thickness
EF: Ejection fraction
FS: Fractional shortening
BW: Body weight
HW: Heart weight
H&E: Hematoxylin and eosin
WGA: Wheat germ agglutinin
NRCMs: Neonatal rat cardiomyocytes
wt: Wild type
mut: Mutated
3′-UTR: 3′-Untranslated region
GO: Gene Ontology
KEGG: Kyoto Encyclopedia of Genes and Genomes
m6A: *N*^6^-methyladenosine
AT1R: Angiotensin type 1 receptor
DKK: Dickkopf
