# Supplementary material for: METTL3 mediates Ang-II-induced cardiac hypertrophy through accelerating pri-miR-221/222 maturation in an m6A-dependent manner
Source: Cell Mol Biol Lett. 2022 Jul 14;27:55. doi: 10.1186/s11658-022-00349-1 (PMC9284900; doi:10.1186/s11658-022-00349-1)
Supplement: Supplementary file 1 — Additional file 1: Figure S1. Identification of knockdown and overexpression efficiency regarding METTL3. NRCMs were transfected with lentivirus vector carrying three different shRNA sequences targeting METTL3. qRT-PCR (A) and Western blot (B) detected the expression of METTL3. n = 3. NRCMs were transfected with plvx-METTL3 vector to overexpress METTL3. qRT-PCR (C) and Western blot (D) detected the expression of METTL3. n = 3. **P < 0.01, ***P < 0.001. Figure S2. The predicted m6A site in sequences of pri-miR-221 and pri-miR-222. Sequences marked in blue and red combined represent the pre-miR-221 (A) and pre-miR-222 (B), while marked in red only as the mature miR-221 (A) and miR-222 (B). The m6A site of pri-miR-221 (A) and pri-miR-222 (B) was marked in green. Figure S3. Evaluate the Wnt/β-catenin in NRCMs transfected with shMETTL3 and miR-221/222 mimics. Western blot detected the expression of β‐catenin and c-Myc (A) in Ang-II induced NRCMs transfected with shMETTL3 and miR-221/222 mimics. n = 3. qRT-PCR detected the expression of c-Myc (B) and DKK2 (C) in Ang-II induced NRCMs transfected with shMETTL3 and miR-221/222 mimics. n = 3. *P < 0.05, **P < 0.01. Figure S4. Identification of knockdown efficiency regarding DKK2. NRCMs were transfected with lentivirus vector carrying three different shRNA sequences targeting DKK2. qRT-PCR (A) and Western blot (B) detected the expression of DKK2. n = 3. ***P < 0.001. Figure S5. Evaluate the effect of DKK2 knockdown on hypertrophy remission induced by miR-221/222 inhibition. qRT-PCR detected the expression of ANP (A) and BNP (B) in Ang-II induced NRCMs with miR-221/222 inhibition and DKK2 knockdown. n = 3. *P < 0.05, **P < 0.01, ***P < 0.001. Figure S6. Evaluate the regulation of METTL3 knockdown on miR-221/222 and Wnt/β-catenin signaling in mouse model. qRT-PCR detected the expression of miR-221 (A) and miR-222 (A) in the three groups. n = 3. Western blot detected the expression of β-catenin (B) in the three groups. n = 3. *P [file 11658_2022_349_MOESM1_ESM.docx]

**Supplementary Materials**

**
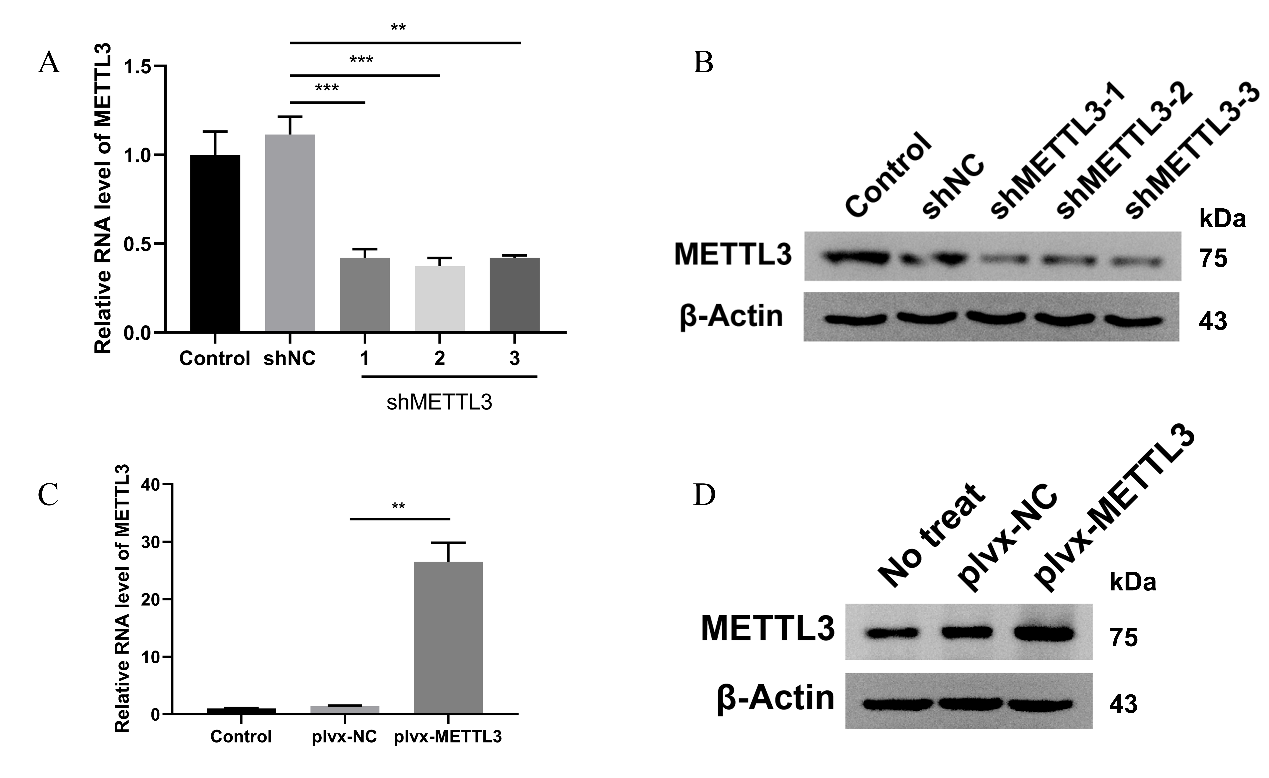
**

**Figure S1.** Identification of knockdown and overexpression efficiency regarding METTL3. NRCMs were transfected with lentivirus vector carrying three different shRNA sequences targeting METTL3. qRT-PCR (A) and Western blot (B) detected the expression of METTL3. n = 3. NRCMs were transfected with plvx-METTL3 vector to overexpress METTL3. qRT-PCR (C) and Western blot (D) detected the expression of METTL3. n = 3. **P < 0.01, ***P < 0.001.

**
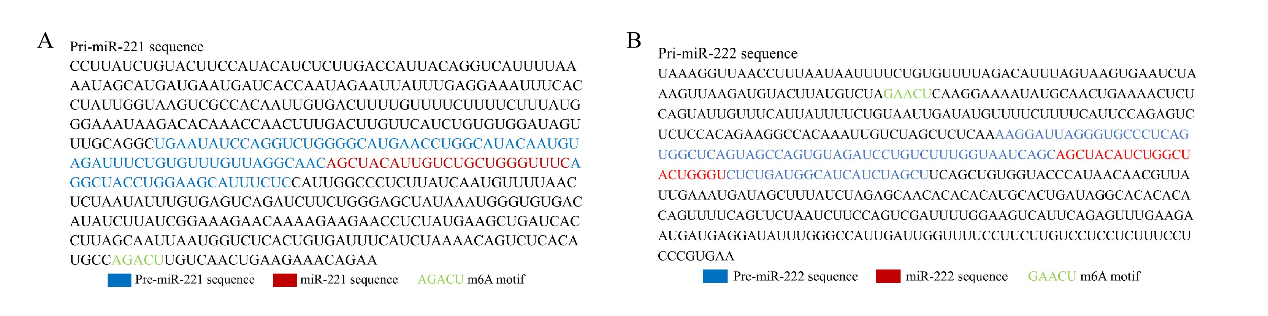
**

**Figure S2.** The predicted m6A site in sequences of pri-miR-221 and pri-miR-222. Sequences marked in blue and red combined represent the pre-miR-221 (A) and pre-miR-222 (B), while marked in red only as the mature miR-221 (A) and miR-222 (B). The m6A site of pri-miR-221 (A) and pri-miR-222 (B) was marked in green.

**
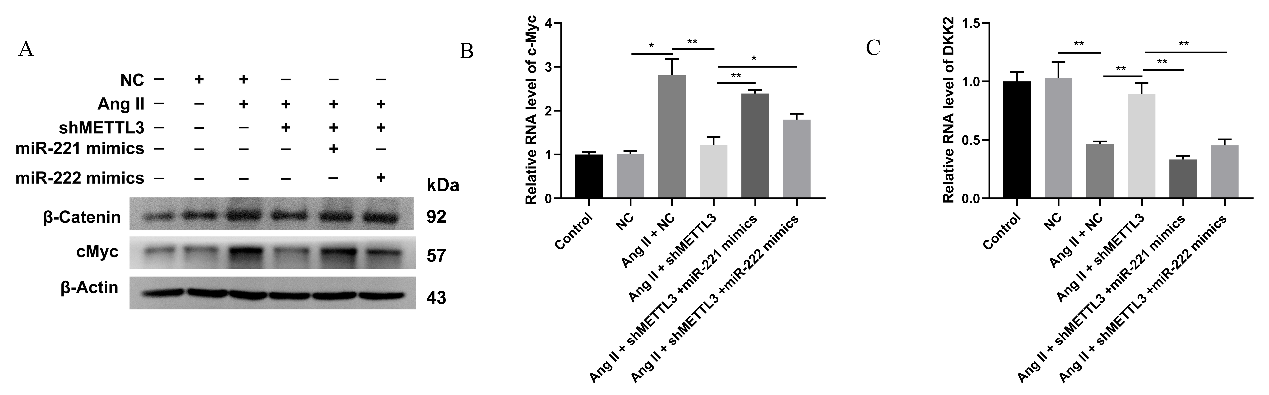
**

**Figure S3.** Evaluate the Wnt/β-catenin in NRCMs transfected with shMETTL3 and miR-221/222 mimics. Western blot detected the expression of β‐catenin and c-Myc (A) in Ang-II induced NRCMs transfected with shMETTL3 and miR-221/222 mimics. n = 3. qRT-PCR detected the expression of c-Myc (B) and DKK2 (C) in Ang-II induced NRCMs transfected with shMETTL3 and miR-221/222 mimics. n = 3. *P < 0.05, **P < 0.01.

**
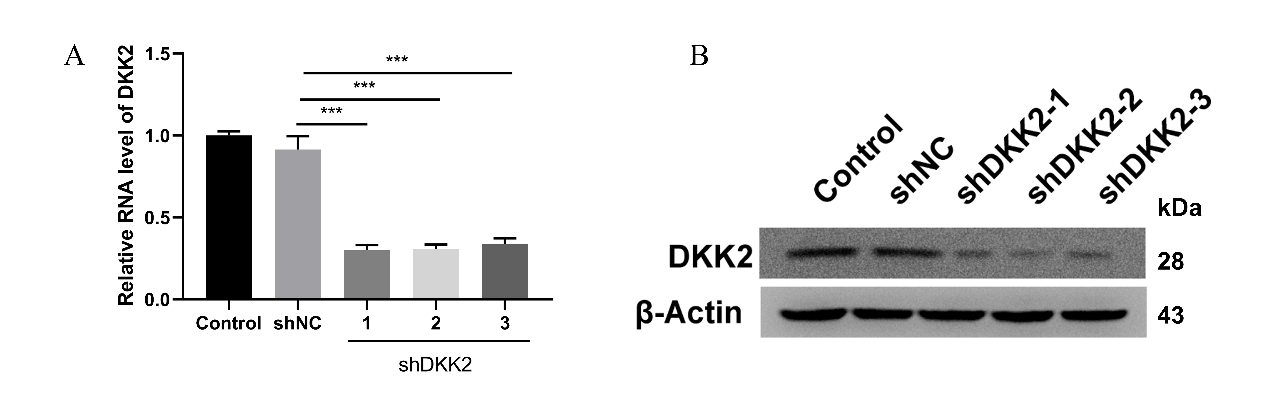
**

**Figure S4.** Identification of knockdown efficiency regarding DKK2. NRCMs were transfected with lentivirus vector carrying three different shRNA sequences targeting DKK2. qRT-PCR (A) and Western blot (B) detected the expression of DKK2. n = 3. ***P < 0.001.


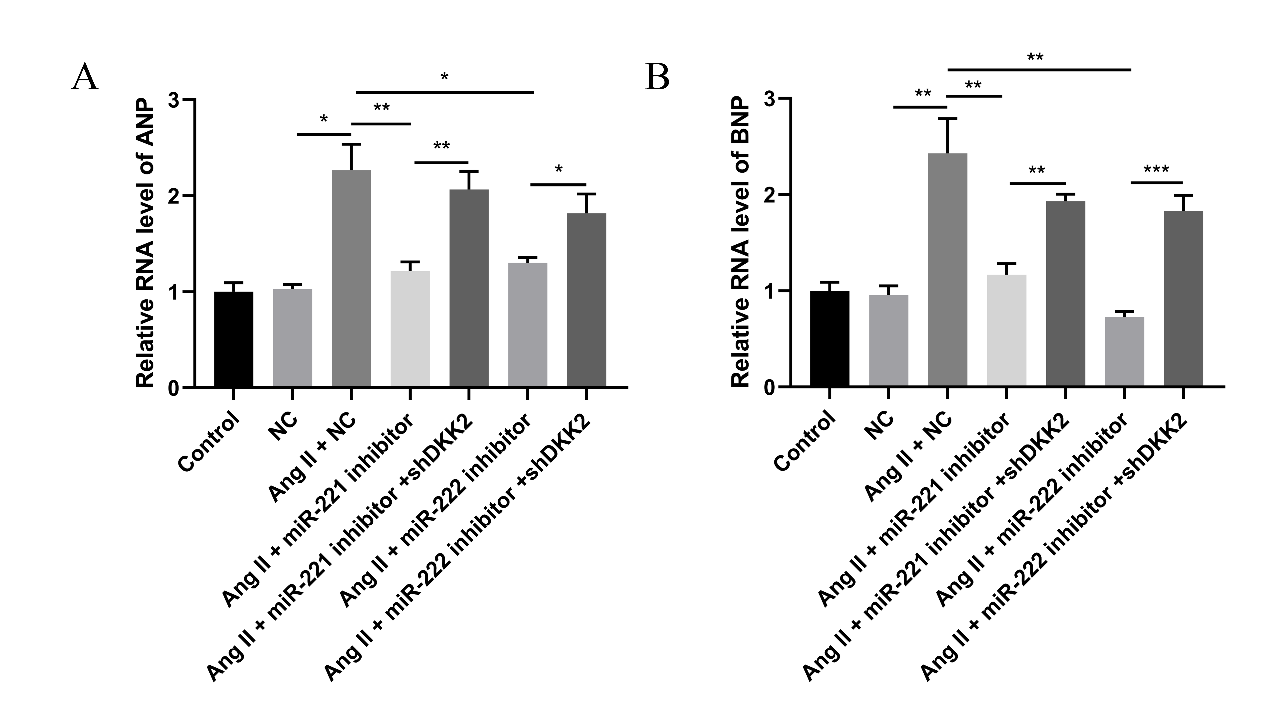


**Figure S5.** Evaluate the effect of DKK2 knockdown on hypertrophy remission induced by miR-221/222 inhibition. qRT-PCR detected the expression of ANP (A) and BNP (B) in Ang-II induced NRCMs with miR-221/222 inhibition and DKK2 knockdown. n = 3. *P < 0.05, **P < 0.01, ***P < 0.001.


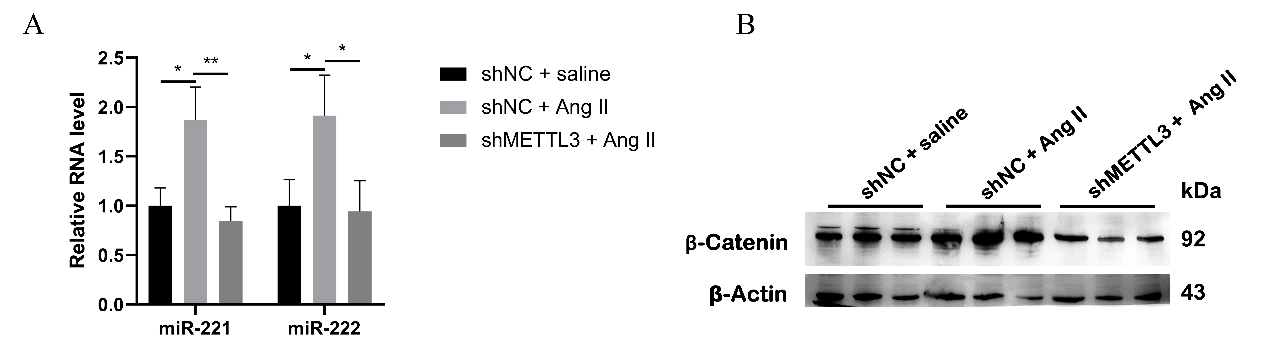


**Figure S6.** Evaluate the regulation of METTL3 knockdown on miR-221/222 and Wnt/β-catenin signaling in mouse model. qRT-PCR detected the expression of miR-221 (A) and miR-222 (A) in the three groups. n = 3. Western blot detected the expression of β-catenin (B) in the three groups. n = 3. *P < 0.05, **P < 0.01.

**Table S1. Sequences of knockdown in Mouse**

| Name |  | Sequences |
| --- | --- | --- |
| shMETTL3 | Sense | 5'-ACCGGAAGCTGCACTTCAGACGAATTTTCAAGAGAAA  TTCGTCTGAAGTGCAGCTTTTTTT-3' |
|  | Antisense | 5'-TCTAAAAAAAAGCTGCACTTCAGACGAATTTCTCTTGA  AAATTCGTCTGAAGTGCAGCTTC-3' |

**Table S2. Sequences of knockdown or overexpression in Rat**

| Name |  | Sequences |
| --- | --- | --- |
| shMETTL3-1 | Sense | 5'-CCGGGCACTTGGACTTAAGGAATCCCTCGAGGGATTCC  TTAAGTCCAAGTGCTTTTTG-3' |
|  | Antisense | 5'-AATTCAAAAAGCACTTGGACTTAAGGAATCCCTCGAGG  GATTCCTTAAGTCCAAGTGC-3' |
| shMETTL3-2 | Sense | 5'-CCGGGGACTATCACAGGGCAGAAACCTCGAGGTTTCTG  CCCTGTGATAGTCCTTTTTG-3' |
|  | Antisense | 5'-AATTCAAAAAGGACTATCACAGGGCAGAAACCTCGAGG  TTTCTGCCCTGTGATAGTCC-3' |
| shMETTL3-3 | Sense | 5'-CCGGGCTGCACTTCAGACGGATTATCTCGAGATAATCCG  TCTGAAGTGCAGC TTTTTG-3' |
|  | Antisense | 5'-AATTCAAAAAGCTGCACTTCAGACGGATTATCTCGAGAT  AATCCGTCTGAAGTGCAGC-3' |
| shDKK2-1 | Sense | 5'-CCGGGGTGGAGAGTTCACAGCTAAGCTCGAGCTTAGCT  GTGAACTCTCCACC TTTTTG-3' |
| shDKK2-2  shDKK2-3  miR-221 inhibitor  miR-221 mimics  miR-222 inhibitor  miR-222 mimics  NC inhibitor  NC mimics | Antisense  Sense  Antisense  Sense  Antisense  Sense  Sense  Antisense  Sense  Sense  Antisense  Sense  Sense  Antisense | 5'-AATTCAAAAAGGTGGAGAGTTCACAGCTAAGCTCGAGC  TTAGCTGTGAACTCTCCACC-3'  5'-CCGGGGGCCAAACTCAACTCCATCACTCGAGTGATGGAG  TTGAGTTTGGCCCTTTTTG-3'  5'-AATTCAAAAAGGGCCAAACTCAACTCCATCACTCGAGT  GATGGAGTTGAGTTTGGCCC -3'  5'-CCGGGGATGGCAGAATCTAGGAAGGCTCGAGCCTTCCTA  GATTCTGCCATCCTTTTTG-3'  5'-AATTCAAAAAGGATGGCAGAATCTAGGAAGGCTCGAGC  CTTCCTAGATTCTGCCATCC-3'  5'- GAAACCCAGCAGACAAUGUAGCU -3'  5'- AGCUACAUUGUCUGCUGGGUUUC -3'  5'- AACCCAGCAGACAAUGUAGCUUU -3'  5'-ACCCAGUAGCCAGAUG -3'  5'-AGCUACAUCUGGCUACUGGGU-3'  5'-CCAGUAGCCAGAUGUAGCUUU-3'  5'-CAGUACUUUUGUGUAGUACAA-3'  5'-UUCUCCGAACGUGUCACGUTT-3'  5'-ACGUGACACGUUCGGAGAATT-3' |

**Table S3. Sequences of Primer for Rat**

| **Genes** | **Sequences** |
| --- | --- |
| **METTL3** |  |
| Forward | 5'-CCGCGCTAGGAACTAGGATG-3' |
| Reverse | 5'-CCACTAGAGGTAGGGGCAGT-3' |
| **pri-miR-221** |  |
| Forward | 5'-GAGAAATGCTTCCAGGTAGCCTG-3' |
| Reverse | 5'-GAATATCCAGGTCTGGGGCATG-3' |
| **pri-miR-222** |  |
| Forward | 5'- GCTAGATGATGCCATCAGAGACC-3' |
| Reverse | 5'-GGATTAGGGTGCCCTCAGTG-3' |
| **miR-221** |  |
| Forward | 5'-GTGCGCAGCTACATTGTCTGC-3' |
| Reverse  RT  **miR-222**  Forward  Reverse  RT  **DKK2**  Forward  Reverse  **c-Myc**  Forward  Reverse  **ANP**  Forward  Reverse  **BNP**  Forward  Reverse  **U6**  Forward  Reverse  **β-Actin**  Forward  Reverse | 5'-CCAGTGCAGGGTCCGAGGTA-3'  5'-GTCGTATCCAGTGCAGGGTCCGAGGTATTCGCACTG  GATACGACGAAACC-3'  5'-GGCGAAGCTACATCTGGCTA-3'  5'-CCAGTGCAGGGTCCGAGGTA-3'  5'-GTCGTATCCAGTGCAGGGTCCGAGGTATTCGCACTG  GATACGACACCCAG-3'  5'-TGTCTGACCCATGAGGGGAA-3'  5'-TAGTGCCAGCATCCATCACC-3'  5'-TGCTCTCCGTCCTATGTTGC-3'  5'-GAGAAGCCGCTCCACATACA-3'  5'-AACCAGAGAGTGAGCCGAGA-3'  5'-CAATATGGCCTGGGAGCCAA-3'  5'-AGCTGCTTTGGGCAGAAGAT-3'  5'-AAAACAACCTCAGCCCGTCA-3'  5'-CTCGCTTCGGCAGCACA-3'  5'-AACGCTTCACGAATTTGCAT-3'  5'-CACTCCAAGTATCCACGGCA-3'  5'-TCCTCCCCTTAGGAGTTGGG-3' |

**Table S4. Sequences of Primer for mouse**

| **Genes** | **Sequences** |
| --- | --- |
| **ANP** |  |
| Forward | 5'-AGACCACCTGGAGGAGAAGATGC-3' |
| Reverse | 5'-GCGAGCAGAGCCCTCAGTTTG-3' |
| **BNP** |  |
| Forward | 5'-TCGGAGGAAATGGCCCAGAGAC-3' |
| Reverse | 5'-AGAGACCCAGGCAGAGTCAGAAAC-3' |
| **β-Actin**  Forward  Reverse  **miR-221**  Forward  Reverse  RT  **miR-222**  Forward  Reverse  RT  **U6**  Forward  Reverse | 5'-TCACTATTGGCAACGAGCGGTTC-3'  5'-CTCCTGCTTGCTGATCCACATCTG-3'  5'- AAGCGCCTAGCTACATTGTCT-3'  5'- CAGTGCAGGGTCCGAGGT-3'  5'-GTCGTATCCAGTGCAGGGTCCGAGGTATTCGCACTGGAT  ACGACGAAACC-3'  5'-AATGCTTAGCTACATCTGGCTAC-3'  5'- CAGTGCAGGGTCCGAGGT-3'  5'-GTCGTATCCAGTGCAGGGTCCGAGGTATTCGCACTGGAT  ACGACAGACCC-3'  5'-GCTTCGGCAGCACATATACTA-3'  5'-CGAATTTGCGTGTCATCCTTG-3' |
